# Bibliographical Department

**Published:** 1880-11

**Authors:** 


### BIBLIOGRAPHICAL DEPARTMENT.

"Nothing extenuate, nor set down aught in malice."

"PREADAMITES; *Or a Demonstration of the Existence of Man Before Adam;* together with a study of their condition, antiquity, radical affinities and progression, dispersion over the earth; with charts and other illustrations. By Alexander Winchell LL. D., Professor ot Geology and Palæontology in the University of Michigan, etc., etc. Second Edition. Chicago: L. L. Griggs & Co. London: Tubner & Co., 1880." We have just risen from the perusal of the foregoing work, for a copy of which we are indebted to the courtesy of the enterprising publishers. The volume is very handsomely gotten up in all respects, and is one of the most fascinating, as well as the most scholarly works we have read for a long while past. In fact, it treats on a subject that so deeply interests all who are fond of thinking at all, that we predict for it an extensive sale. We have long been fully convinced of the existence of races of men before Adam, and of races that were contemporaneous with his family, and have been accustomed to speak and write of Adam as the representative head of the Jewish race; but we had not thought that he was descended from one of the pre-existing or contemporaneous races, and certainly not from an inferior, and least of all, from *negro* ancestry! Indeed, we have been fully convinced of the *plural origin* of the races for more than thirty years.

"HYGIENE AND SANATIVE MEASURE FOR CHRONIC CATARRHAL INFLAMMATION OF THE NOSE, THROAT AND EARS, Part 1. By Thomas F. Rumbold, M. D., St. Louis, Geo. O. Rumbold & Co., 1880."

We are in receipt of a neat little volume of 179 pages, bearing the above title page. It is written in plain, practical style, and shows that the author intended it to be useful rather than ornate in detailing his experience and suggestions on the subject discussed in his book. His remarks on the use of tobacco, are judicious, and if followed, cannot fail to prove beneficial, not only in the class of troubles in which its use is interdicted, but in all other conditions of the system, whether in health or disease. Part 2 will be devoted to the treatment of the diseases the doctor has made a specialty of for many years. It would be impossible to conjecture the value of his therapeutic resources and directions, but if they should contain the amount of practical common sense to be found in the little volume before us, the completed work will not fail to be useful to the profession.

NATIONAL ASSOCIATION FOR THE PROTECTION OF THE INSANE, *And the Prevention of Insanity*, Boston, 1880.—*Paracentisis pericardii, and a New Method of arresting puerperal eclampsia.* Papers read before the Iowa State Medical Society. By George M. Staples, M. D., of Dubuque.

*Bulletin du Canal Inter-Oceanique Paraissant Le 1 Et Le 15 De Chaque Moiso Vendredi 1 Octobre, 1880. A Paris.*

ROCKY MOUNTAIN MEDICAL REVIEW.—A Monthly Journal of Scientific Medicine and General Science, edited by A. Wellington Adams, M. D., with four Associate and one Assistant Editors, Vol. I, No. 1, September, 1880. Colorado Springs, Col. We notice among the Associate Editors the name of Dr. B. P. Anderson, a highly intelligent and valued friend, and whilom pupil. This journal is well printed and contains several articles of value to the profession. We wish the enterprise much success.

THE SPECIALIST AND INTELLIGENCER, A Monthly Journal of Medical Science, devoted specially to the publication of original and selected articles on Diseases of the Eye, Ear, Throat and Skin, Venereal Diseases, etc., Vol. I, No. I, edited by Charles W. Dulles, M. D., Philadelphia. $1.50 per annum. Its articles will be found valuable to specialists.

THE PHYSICIANS' VISITING LIST.—We are indebted to Messrs. Lindsay & Blakiston for a copy of their valuable visiting list for 1881. Almost every physician in this country at least, is so well acquainted with this convenient and excellent pocket companion that it is only necessary to say it is out again in all its accustomed neatness, to induce them to supply themselves with a copy.

*Annual Announcement Savannah Medical College*, for session of 1880-81. Has a full faculty, and good clinical facilities.

The Monthly Review of Dental Surgery, being the Journal of the British Dental Association, edited by Alfred Coleman, F. R. C. S., L. D. S., 22 Grosvener street, Grosvener Sqr., London. Price six pence. Is a most excellent periodical, and it is with much pleasure that we add it to our exchange list.

